# Development and Validation of the RAFFLE: A Measure of Reasons and Facilitators for Loot Box Engagement

**DOI:** 10.3390/jcm10245949

**Published:** 2021-12-18

**Authors:** Joanne Lloyd, Laura Louise Nicklin, Stuart Gordon Spicer, Chris Fullwood, Maria Uther, Daniel P. Hinton, Jonathan Parke, Helen Lloyd, James Close

**Affiliations:** 1 School of Psychology, Faculty of Education, Health and Wellbeing, University of Wolverhampton, Wolverhampton WV1 1LY, UK; C.Fullwood@wlv.ac.uk (C.F.); M.Uther@wlv.ac.uk (M.U.); D.Hinton@wlv.ac.uk (D.P.H.); 2 Institute of Education, Faculty of Education, Health and Wellbeing, University of Wolverhampton, Wolverhampton WS1 3BD, UK; Laura.Nicklin@wlv.ac.uk; 3 School of Psychology, Faculty of Health, University of Plymouth, Plymouth PL4 8AA, UK; Stuart.Spicer@plymouth.ac.uk (S.G.S.); helen.lloyd-1@plymouth.ac.uk (H.L.); James.Close@plymouth.ac.uk (J.C.); 4 Sophro, Newark Beacon Innovation Centre, Cafferata Way, Newark NG24 2TN, UK; DrjParke@yahoo.co.uk

**Keywords:** lootboxes, video gaming, motivations, motives, microtransactions, gambling, addiction, scale development, scale validation

## Abstract

Qualitative studies have identified a diverse array of motivations for purchasing items within video games through chance-based mechanisms (i.e., “loot boxes”). Given that some individuals—particularly those at risk of disordered gaming and/or gambling—are prone to over-involvement with loot box purchasing, it is important to have a reliable, valid means of measuring the role of different motivations in driving purchasing behaviour. Building on prior qualitative research, this paper reports the development and validation of the “RAFFLE” scale, to measure the Reasons and Facilitators for Loot box Engagement. A 23-item, seven-factor scale was developed through cognitive interviews (*n* = 25) followed by two surveys of UK-based gamers who purchase loot boxes; analysed via exploratory (*n* = 503) and confirmatory (*n* = 1495) factor analysis, respectively. Subscales encompassed “enhancement’; “progression’; “social pressure’; “distraction/compulsion’; “altruism’; “fear of missing out’; and “resale”. The scale showed good criterion and construct validity (correlating well with measures of loot box engagement; the risky loot box index (*r* = 0.63) and monthly self-reported spend (*r* = 0.38)), and good internal validity (Cronbach’s alpha = 0.84). Parallels with, and divergence from, motivations for related activities of gaming and gambling, and alignment with broader theoretical models of motivation, are discussed.

## 1. Introduction

Video gaming (hereafter, “gaming”) is an activity in which over half of the UK population are estimated to take part, generating billions of pounds in annual revenue [1]. Within most video games (across desktop, console, and mobile platforms), players can purchase digital items to use, wear, or display in-game, which have aesthetic and/or functional value to the player [2]. In addition to selling outright at a fixed cost, items may also be obtained through obtaining and opening a “loot box”—a container whose digital contents vary in monetary or subjective value and are not revealed until after purchase [2]. Loot boxes, while they can sometimes be “earned” through in-game tasks, are typically purchasable (i.e., require monetary “stake”), and yield items that range from essentially worthless losses to highly prized wins, according to a variable reinforcement schedule. This has led to suggestions that they are “psychologically akin to gambling” [3], and they have attracted much attention over the past decade from the media, policymakers, and academics (e.g., [4,5]). Systematic review evidence has established that people experiencing problematic gambling and/or video gaming show significantly greater engagement with loot boxes than people scoring below threshold for problematic gaming/and or gambling [5,6,7,8]. Furthermore, loot box purchasers tend to be from those demographic groups that are more at risk of addictive behaviours and associated harm [9].

It is important to understand not just who buys loot boxes and what co-occurring problems they may be experiencing, but also the reasons why people purchase them. Identification of the motivations for engaging in addictive behaviours has the potential to inform educational messaging and interventions [10,11]. Furthermore, motivations for taking part in psychologically “rewarding” activities such as gambling vary across individuals [12], and where particular motivations are disproportionately associated with problematic levels of engagement or with other comorbid conditions (as has been seen, for example, with gambling [13]), an understanding of the drivers of participation could help identify those at particular risk of harm.

A small number of studies have begun to unpick the motivations for purchasing loot boxes. In an online survey, Zendle and colleagues [14] asked a sample of adolescent loot box buyers to identify their reasons for purchase, via brief free-text responses. The 441 responses were categorised into eight motivations; “for gameplay advantages”; “to gain specific items and create a collection”; “the fun, excitement and thrills of opening the box itself”; “appearance reasons”; “support the developers or pay for the game”; “the perception that loot boxes are good value”; “time advantages”; and “profit” [14]. Nicklin and colleagues conducted a series of in-depth qualitative interviews with adult gamers about their reasons for purchasing, and identified seven themes [15]. These were broadly consistent with Zendle et al.’s findings: “the opening experience” (synonymous with Zendle et al.’s motive around fun and excitement of opening the box); “the value of box contents” (which covered desire to win items with aesthetic, functional and/or monetary value); “game-related elements” (which encompassed the gameplay advantages factor identified by Zendle and colleagues but also included desire to speed up/facilitate progression, including in single player games, rather than being only about advantages over other players); social influences (which overlapped with Zendle et al.’s “appearance” motive and also their “supporting developers” motive, but also encompassed broader socialisation motives); emotive/impulsive influences (i.e., feeling compelled to purchase boxes, sometimes as an escape from boredom or negative feelings); fear of missing out (on shared experiences or on limited time offers); and external triggers/facilitators (such as promotions or events—overlapping with Zendle et al.’s motive of perceptions of “good value”).

These two studies evidence a wide range of motivations for engaging with loot boxes; some closely aligned with gaming involvement (e.g., seeking items to afford gameplay advantages), and others more closely aligned to gambling-related motives (e.g., seeking fun or excitement from opening the box)—as might be expected, given the established links between both gambling and gaming, and loot box purchasing. Notably, purchasers reference both external variables—such as events and promotions, and exciting sounds and animations—and intrinsic drivers, centring around fulfilling desires or satisfying needs, such as the desire to be competitive, or to experience excitement. This is consistent with the literature on motives for both gaming and gambling, which are influenced by external drivers such as advertisements and promotions [16,17] and the structural characteristics of the game [18,19], in addition to being behaviours people pursue in order to satisfy psychological needs for competence, relatedness, or autonomy (which are often framed within concepts of self-determination theory, [20,21,22]).

While these qualitative studies provide a detailed account of individuals’ varied reasons for purchasing loot boxes, to date, there is no existing, validated scale to measure motivations quantitatively. Despite parallels with gaming and gambling, existing scales for these activities are not directly transferable to loot box purchasing; a unique activity that merits a bespoke scale. Factors such as gaming as a means of “violent catharsis” [23] or for “opportunities to lead” [24], for instance, are not likely to be directly relevant to loot boxes. This paper reports the development of a scale to measure “Reasons And Facilitators For Loot box Engagement” (hereafter, “the RAFFLE”), with candidate items derived from the data collected via qualitative work described above [15].

This scale was developed and validated in three phases, described below: (1) cognitive interviews, to refine items developed from qualitative interview data; (2) an online survey analysed by exploratory factor analysis and an expert opinion panel, to refine scale content, identify factor structure and test internal consistency and construct validity; and (3) a large-scale validation survey analysed by confirmatory factor analysis, to test factor structure, internal consistency and construct validity.

## 2. Study 1: Cognitive Interviews for Item Refinement

This study was conducted to refine the content and wording of the initial pool of items, which were developed via in-depth qualitative interviews (reported in [15]). The aim was to maximise the clarity and appropriateness of the candidate scale items for study 2 by identifying problematic items for removal or editing; and adding novel items or wording where required.

### 2.1. Materials and Methods (Study 1)

Twenty-five gamers aged between 16 and 44, who had bought loot boxes at least once, were purposively recruited from across the UK to ensure a range of demographic characteristics including age, sex, ethnicity and geographical region. Nineteen males and six females with a mean age of 25.4 years (SD = 6.93), took part between July and August 2020. Participants were recruited from rural and urban areas across the North, Midlands, and South of England, and while the sample was primarily White British in ethnicity, there were participants of Black, Asian and mixed ethnicities. A further 15 experts by experience (11 males and 4 females who had bought loot boxes within video games) attended one of 5 online stakeholder involvement workshops.

An initial pool of 55 items was developed by an expert panel of 9 academic researchers (authors of this paper), based primarily on findings from 28 in-depth qualitative interviews (reported in [15]), and supplemented by existing literature (e.g., [14]). At least two items relating to each of the seven themes identified from the qualitative interviews were incorporated into the item pool, along with items probing additional factors identified through literature review or the aforementioned public involvement workshops, where attendees were invited to critically assess our candidate items. Approximately half of the items were prefaced with the text: “When you buy a loot box for real money, how often does each of the following reasons influence your purchase?” with response options of “never’, “occasionally’, “often”, and “always”. The other half were prefaced with the text: “When you buy a loot box for real money, to what extent do you agree that each of the following reasons influences your purchase?” with response options of “strongly disagree’, “disagree’, “agree”, and “strongly agree”. In addition to gaining participant feedback on the items themselves, respondents were asked which question wording and response scale they preferred. This question was also asked at the stakeholder workshops, for additional feedback.

After being presented with an online information sheet and providing digital informed consent (via checkboxes within Qualtrics online survey software), participants were interviewed remotely (due to COVID-19 restrictions) by researchers LLN or SGS, with interviews lasting approximately 1 h, on average. They were presented with each of the 55 candidate scale items, and explicitly instructed to “think aloud” and explain their thought process in responding to the item. This approach was coupled with verbal probing techniques to enquire about language and clarity, and the item’s perceived relevance and importance (as per established cognitive interviewing protocols [25]). For instance, after a participant read out an item, spoke their thoughts aloud, and said what their response would be, the interviewer prompted (as and when appropriate) with questions such as “how did you decide what response to give?”; “does that question make sense to you?” and “could the wording be improved to make it easier to understand?”. Interviews were recorded for ease of reviewing the responses, and detailed notes on participant responses and suggested edits for each of the questions were captured in a spreadsheet.

After each 4–6 interviews, meetings with the wider research team took place where items were refined iteratively based on feedback from batches of interviews, with all changes to the wording, however minor, logged in a new column of the tracking spreadsheet. Subsequent batches of participants reviewed the revised items in an iterative process, to ensure that after being adapted in light of participant feedback, newly revised items were member-checked, rather than simply assuming that the research team’s changes had effectively addressed issues.

### 2.2. Results (Study 1)

Table 1 summarises how the original candidate items—shown in the left-hand column, were either retained in their original format (where deemed appropriate, relevant and clear); dropped altogether (where highlighted as being irrelevant); re-worded (where identified as ambiguous or lacking clarity); or merged with other items (where judged to be redundant), to form the new 39-item longlist presented in the right-hand column, to be used in the subsequent validation survey. The response options of “never”, “sometimes”, “often”, “always” were preferred by over half the participants, who considered them the clearest and most appropriate for quantifying the frequency with which each potential motivation influenced loot box purchasing.

## 3. Study 2: Validation Survey 1, EFA, and Expert Opinion Panel

We were able to draw on the literature on motivations for gaming and gambling to give a broad sense of what kinds of subscales or “domains” our candidate items might fall into; for instance, we expected that there would be motivations around fun or excitement; around social factors; and around in-game progression. However, motivations for loot box purchasing specifically have only been studied in a few recent studies, and our qualitative work indicated that they are not identical to motives for either gaming or gambling alone, and are nuanced and overlapping. For instance; buying loot boxes to try and obtain items that will allow you to catch up with friends, is a novel and loot-box specific motivation, and it was unclear whether this would load onto a “social” or a “progression/competition” related factor. Therefore, we utilised an “exploratory factor analysis” for the first phase of validation, to examine the scale’s factor structure and identify possible subscales in a data-driven manner without pre-imposing an a priori theoretical framework. This phase also facilitated item reduction, identifying poorly performing and/or redundant items for removal, and provided an opportunity to test the scale’s internal consistency and construct validity.

### 3.1. Materials and Methods (Study 2)

Between 17 November 2020 and 27 November 2020, 503 participants were recruited through Prolific Academic. Those who incorrectly answered one (*n* = 11) or both (*n* = 7) attention checks (e.g., “this is an attention check; please select “strongly disagree” for this item”) were removed due to risk of non-serious responding, leaving 485 participants. Their mean age was 31.66 (SD 10.90; range 18–80); 50.7% were female and 49.3% were male.

Participants were asked to rate the frequency of each reason for buying loot boxes of the 39 longlist items (right-hand column of Table 1), as, “never’, “sometimes’, “often”, or “always”. We also asked about spend on loot boxes (“thinking about the past year, how much money did you spend in a typical month on loot boxes?”), and administered the Risky Loot Box Index (RLI; [26])—a 5-item measure of problematic loot box involvement (with items such as “once I open a loot box, I often feel compelled to open another’; Cronbach’s alpha for this scale in the current study was 0.792). These were included to allow us to examine the scale’s “construct validity”: if it is effective at measuring motivations for buying loot boxes, a correlation would be expected with these other measures of loot box engagement.

#### Analysis

Participants who wished to complete the survey were informed that the study required them to respond to all items. All participants complied with these instructions; therefore, there were no missing data. All scores were within 1.1 standard deviations from the mean, and most variables had skewness indices within the “acceptable” range of −2 to +2; [27]; see Table 2. Three items (about pressure from friends, fear of shame, and influence of streamers) were skewed and showed evidence of kurtosis (as only a small (<10% proportion) of the sample endorsed these motivations any more than occasionally); but given the possibility that skewed items may nevertheless be important as potential predictors of more extreme engagement, we included them in the EFA. Normal distribution is not an assumption for principal axis factoring, as used in this analysis, nor for the confirmatory factor analysis based on polychoric correlations between categorical variables, conducted in the subsequent (study 3) analysis. Examination of bivariate correlations indicated no problems of multicollinearity (all values of *r* were <0.63).

We used principal axis factoring with direct oblimin rotation (due to expectation of some intercorrelation between resulting factors, delta was set at 0). All 39 items were entered with “eigenvalues over 1” as the initial extraction technique. All communalities were >0.2.

Items were removed stepwise, beginning with the “poorest’—based on “loadings” on the pattern structure matrix, and/or cross-loading. Candidates for removal were identified through loadings >0.3 on more than one item, and/or <0.2 difference between loadings [28], with the model re-run after each removal. This iterative process allows the factor solution to be monitored at frequent intervals (useful, as each removal will impact the remaining items [29]). Table S1 (Supporting Information) details the order of item removal and rationale for each removal. An expert opinion focus group (*n* = 7 academics with expertise in measurement science, gambling and gaming) further informed decisions about order of item removal through a combination of numeric ratings and qualitative comments. Item removal proceeded until a stable solution (with no items having primary loadings of <0.4, or cross-loadings of >0.3 on multiple items on the pattern matrix) was reached. At this point, eigenvalues and the scree plot were examined, and considered alongside interpretability of the factor structure, to determine whether the number of extracted factors was optimal.

### 3.2. Results (Study 2)

Descriptive statistics for the 39 original candidate items are presented in Table 2. After 16 item-removal iterations, a stable solution was obtained in the form of a 23-item, seven-factor scale (KMO = 0.849, and Bartlett’s test of sphericity <0.0001). The seven-factor solution explained 63.7% of the variance, and there was a clear case for selecting seven factors, based on interpretability (all factors made theoretical sense and had clear strong loadings and good subscale Cronbach’s alphas), and the fact that the eigenvalues of all seven factors were over 1, and they dropped sharply (as seen also from inspecting the elbow of the scree plot) to 0.80 for the 8th (unretained) factor.

Table 3 and Table S2 show the pattern and structure matrices, respectively, for the resultant solution. The full, 23-item scale had a Cronbach’s alpha of 0.856, indicating good reliability, and individual factors also had adequate to good reliability (0.60 to 0.82), as summarised alongside communality coefficients for each item; eigenvalues for each factor prior to rotation; and percentage of variance explained by each factor, in the bottom three rows of Table 3. We considered the alpha values between 0.6 and 0.7 to be acceptable given that these were factors containing few (2–3) items.

#### 3.2.1. Factor Interpretation

Factor 1, “enhancement”, which explained the most variance (over a quarter) and had the strongest reliability, contained items relating to enjoyment/enhancement. Some items reference enjoyment of opening the boxes and how that makes the opener feel, while some refer to enjoying or valuing the items within the boxes, but the common thread is the seeking of pleasure as a motivating force.

Factor 2, “progression”, contained items related to the desire to progress within a game—i.e., to come back from a failure or defeat; to overcome a hurdle; or to gain an advantage of some kind to facilitate progression.

Factor 3, “social pressure”, contained items relating to (negative) social pressure, referring to fear of being shamed or left out, and to direct pressure from peers.

Factor 4, “distraction/compulsion” contained items relating to feeling compelled to purchase (“can’t stop” and “urge”) and to being motivated by a desire to take one’s mind off their life or relieve boredom.

Factor 5, “altruism”, comprised just two items which referred to altruistic motives, specifically, the supporting of good causes and of games developers.

Factor 6, “fear of missing out” was comprised of three items referring to concerns about missing out on something—from items in a collection to an in-game event or a special offer.

Factor 7, “resale”, was comprised of two items, both of which were concerned with the desire to obtain items to sell on, either for real or in-game currency.

#### 3.2.2. Criterion-Related Validity

Table 4 presents Spearman’s rho correlations between the RAFFLE (as an overall score, and as subscale scores), risky loot box involvement (RLI score), and monthly loot box expenditure. All were highly statistically significant—particularly correlations between RLI and the overall RAFFLE score, and between the RLI and the distraction/compulsion subscale score, indicating good construct validity.

## 4. Study 3: Validation Survey 2 and CFA

As part of a comprehensive online survey reported elsewhere (Close et al., forthcoming), a sample of UK-based loot box purchasers completed the refined scale developed in study 2, and a CFA was conducted to test the fit of the factor structure identified in study 2 in a larger sample, and to provide another opportunity to examine the scale’s internal consistency and construct validity.

### 4.1. Materials and Methods (Study 3)

Between 8 March 2021 and 31 March 2021, 3063 participants were recruited from Prolific Academic. After the removal of 71 response sets due to failing one or more attention checks—54 who either did not complete all questions and/or revoked consent, and 220 who stated at screening that they did not purchase loot boxes, but later gave a non-zero answer to monthly spend—2718 remained. Of these, 1495 had experience of purchasing loot boxes. Of these, 51.5% were female, 47.6% male, and 0.9% non-binary/third gender. Mean age was 31.16 (SD 9.93) years (range: 18–69 years).

Participants who bought loot boxes completed the 23-item scale developed during validation survey 1. Response categories were identical to those used in validation survey 1, and again we administered the RLI [26] and question about loot box spend.

#### Analysis

Confirmatory factor analysis was performed with MPlus [30], using diagonally weighted least mean squares (as advised for use with Likert-scale responses [31,32]). We pre-specified the structure of subscales identified in validation survey 1 (as shown in Table 3), and metrics were compared against goodness-of-fit benchmarks recommended in Hooper et al. [32]. Scale reliability was evaluated using Cronbach’s alpha, and criterion validity was examined by correlating RLI and monthly loot box spend with RAFFLE scale and subscale scores. Appropriateness of the scale structure was evaluated based on these metrics, but also based on theoretical underpinnings, by an expert opinion panel (comprised of the authors of this paper).

### 4.2. Results (Study 3)

#### 4.2.1. Confirmatory Factor Analysis

Fit indices are summarised in Table 5. Chi-square was highly significant (*X*^2^ = 18,948.52, df = 253, *p* < 0.0001), indicating divergence of what was observed from the predicted model. However, due to this metric’s sensitivity to sample size, this is typical in large samples—where other metrics are more informative [32].

According to the Standardised Root Mean Square Residual value—which Kline deems to be the most appropriate metric for Likert-type response scales—the scale’s structure was a good fit (scoring 0.066; values should be below 0.08 [31]). The scale’s performance on other metrics were reasonable; root mean square error of approximation (RMSEA) was 0.08 (the benchmark being <0.08 [31], Comparative Fit Index (CFI) was 0.89 (the benchmark being >0.9 [31] and the Tucker–Lewis Index was 0.87 (where it should, ideally, be >0.9 [31]). This was not unexpected, as otherwise robust scales often fall slightly short of such cut-offs when conducting CFA in applied research—particularly when structure is complex and there is some degree of intercorrelation between subscales [33].

#### 4.2.2. Reliability and Criterion Validity

Cronbach’s alpha for the entire scale was very good (0.84), and for the subscales was good (0.69 to 0.79), with the exception of the “resale” motives subscale—which fell just under the acceptable threshold of 0.6, which is not unusual for a subscale comprised of only two items—furthermore, inter-item correlations were within the optimal range (*r* = 0.394).

As in validation study 1, we observed highly significant correlations (*p* < 0.001) between overall RAFFLE scores, and scores on each subscale, and both of the measures of loot box involvement (i.e., RLI scores and self-reported monthly spend). As summarised in Table 6, these correlations were all statistically significant, but strength of correlation varied from weak (*r* = 0.10 for “altruism” subscale and RLI score) to moderate (e.g., *r* = 0.47 for the “enhancement” subscale and RLI score) and strong (*r* = 0.59 for the distraction/compulsion subscale and RLI score). The total RAFFLE score which had a 0.63 correlation with RLI and 0.38 correlation with spend—indicating strong criterion validity.

#### 4.2.3. Measurement Invariance

Measurement invariance across both gender and age was explored by comparing fit indices for configural, metric and scalar models (where Δ CFI should be lower than 0.01 and Δ RMSEA should be lower than 0.015 [34]). With respect to gender, full measurement invariance was found based on comparison of the configural (CFI = 0.897; RMSEA = 0.078), metric (CFI = 0.896; RMSEA = 0.077) and scalar (CFI = 0.893; RMSEA = 0.074) models. To investigate measurement invariance with respect to age, the sample was first divided into two groups based on a median split at the median of 30 years. Full measurement invariance was found based on comparison of the configural (CFI = 0.899; RMSEA = 0.076), metric (CFI = 0.895; RMSEA = 0.077) and scalar (CFI = 0.895; RMSEA = 0.073) models.

## 5. Discussion

The aim of this work was to develop and validate a scale to measure reasons and facilitators for purchasing chance-based items within video games. We were motivated by the lack of pre-existing scales specific to loot box purchasing, despite the existence of multiple scales to measure gaming and gambling motives.

Following previous qualitative work that identified a broad array of factors that drive people to purchase loot boxes [15], our cognitive interviews and two validation surveys yielded a 23-item scale (the “RAFFLE”), with seven subscales, containing between two and six items each, which encompassed “enhancement’; “progression’; “social pressure’; “distraction/compulsion’; “altruism’; “fear of missing out’; and “resale’.

Most of these subscales have strong parallels with those seen in existing measures of motivations for other activities. “Enhancement” is one of the most universal: factors to do with fun, excitement, or recreation have been identified across numerous studies of motivations for both gambling [13,35,36,37,38,39] and gaming [40,41,42,43,44], and have generally been found not to correlate strongly with problematic involvement [13,45]. Within self-determination theory, fun and excitement are defined as types of “intrinsic motivations”; people who find an activity fun or exciting are likely to participate in it because it is enjoyable to them, in and of itself [46]. Within the RAFFLE, the enhancement factor covers enjoyment of opening loot boxes, and how that makes the opener feel (specifically, excitement, fun, and the feeling of winning), along with enjoyment of the items within the boxes (because they are visually appealing; perceived as valuable; or make the opener feel good about themselves). All items within this subscale refer to the promise of reward or pleasure of some kind as prompting purchasing. Interestingly, this factor is the only one that contains items explicitly referring to the element of “surprise”, which is one of the prominent commonalities between gambling and loot box opening (and less obviously associated with gaming).

In contrast, the second factor, “progression” has many parallels in the literature on gaming motivations, where motives to do with achievement [47], competition and progression [41] and competence [48] are widely reported. A smaller number of gambling motivations studies have also reported desire for a sense of competence as a driver [36,49]. These motives have also been interpreted through the lens of self-determination theory, whereby gaming or gambling can be perceived as a means of meeting core psychological needs, including the need for competence [36,48]. This aligns with the idea that individuals should be appreciated as active “users” of games, rather than passive consumers who games are “done to” [44]. It is also consistent with the “uses and gratifications” theory of media use which has been applied to both gaming [41] and to in-game purchases [50], and focuses on how the positive benefits (be they practical or psychological) derived from that activity are important in promoting engagement. Competence-based gratifications could be described as central to the RAFFLE progression factor, which encompasses motivations to buy loot boxes to overcome challenges within the game itself, and to gain a competitive edge over others.

Factor 3, “social pressure” has parallels with social motives for gaming and gambling. However, this factor encompassed negative social pressure, with items referring to fear of being shamed or left out, and to direct pressure from peers. Social motivations in the wider literature often encompass more positive factors, such as opportunities for teamwork or for forming relationships as motivators for gaming [41,47] and gambling [22]. While our qualitative work did highlight that some gamers participated in loot-box openings as a pleasant, shared social experience [15], the scale development process identified a prominent social motive that was more negatively framed. Given that peer pressure and perceptions that gambling is a social norm are both prospectively associated with problematic gambling [51], future research should investigate whether this motivation for loot box purchasing is salient in predicting problematic engagement. Social motives can also be understood in relation to self-determination theory, as the desire to fulfil the need for “relatedness”, and while our social pressure subscale is primarily about avoiding negative social feedback, this could nevertheless be perceived as important, by some players, to gain or maintain relatedness.

Factor 4, which we named “distraction/compulsion”, contained items relating to feeling compelled to purchase (“can’t stop” and “urge”). This was the most strongly correlated of all the factors with RLI scores, as might be expected given the fact that two of the four items pertain to feeling out of control of purchasing. While the RLI items focus primarily on indicators of current harmful engagement, however (such as playing longer than intended and putting off other activities), the distraction/compulsion subscale items focus on the feelings that drive purchasing. In particular, desire to relieve boredom and take one’s mind off one’s day-to-day life loaded onto this factor. Motivations around boredom, escapism and coping with day-to-day life have been frequently observed in the contexts of both gaming [44,52,53] and gambling [13,38,42]. Some studies have found that “escapism” can be a relatively benign motivation, reported by those who are at low risk of problematic gambling [54,55], but others have identified escape and coping based motivations as being particularly characteristic of problematic gamblers [12]. This may be due to interactions between escape-based motivations and the existence of mood-related difficulties or environmental stressors. Within the gaming literature, multiple studies have found evidence of such an interplay [56,57]. While escapism can be experienced as a positive affordance of gaming for some; others (i.e., those experiencing difficulties of some sort in their “real lives”) appear to be vulnerable to over-involvement in the activity when they participate in search of escape [56].

Factor 5, labelled “altruism”, consisted of only two items, referring to purchasing loot boxes to support good causes and games developers. While they are not as universally reported as other motives, charitable motives have been identified within the gambling literature [58], and “supporting the developer” was reported as a motive by around 10% of adolescent loot box purchasers [14]. It is beyond the scope of this study to determine whether there were hidden motives underpinning these self-reported charitable motivations for loot box purchasing in our sample, but there are theories that charitable motives can be used as “alibis” for engagement in stigmatised activities [59]. However, the fact that this subscale had the lowest correlation of all with risky loot box engagement suggests that it is not likely to be a “cover” for riskier motivations, and may be one of the most benign reasons for purchase.

Factor 6 was comprised of three items referring to how purchasing was driven by concerns about missing out on something—from items in a collection to an in-game event or a special offer; and was named “fear of missing out”. Zendle and colleagues accurately predicted that the “limited time” nature of many loot boxes could be an important factor in their appeal [14], and fear of missing out (“FoMO”) has been studied extensively as a motivator of engagement with online activities—particularly social media [60], but has also more recently been studied in a gaming disorder context [61]. However, the “FoMO” literature focuses on fear of missing out on social experiences, whereas these items in the RAFFLE encompass fears about missing out on obtaining digital items—so for the avoidance of confusion, we do not use the “FoMO” abbreviation here. Importantly, our “fear of missing out” factor captures the importance of the external environment, and how features of this can impact upon the individual, tempting them to engage. Empirical observational and/or experimental studies can offer a valuable means of establishing how different characteristics of the environment (including advertising and promotions) impact upon decisions to purchase, as they have in gambling research [62,63]. However, this factor on a self-report scale provides a person-centred insight into the extent to which the individual perceives an influence of these external environmental features on their purchasing decisions. Consistent with findings from the gambling literature that moderate-risk gamblers were particularly susceptible to time-limited bet offers [62], scores on this subscale had a moderately high correlation with RLI scores, suggesting that those susceptible to it may be at somewhat elevated risk of engagement.

Finally, factor 7, labelled “resale”, was comprised of just two items, both of which were concerned with the desire to obtain items to sell on, either for real or in-game currency. The parallels with monetary gambling motivations—identified in many studies of gambling motives—are clear [35,36,64]. The self-determination theory lens has also been applied to monetary motivations for gambling, wherein they are conceptualised as “extrinsic motivations” [36], i.e., underpinned by a goal or reward beyond the inherent enjoyment of the activity. It is notable that this factor explained the least variance of all seven. This aligns with findings that monetary gain is a relatively niche motive for loot box engagement, reported by less than 1% of a sample of adolescent gamers [14], and with the fact that links between gambling and loot box purchasing are not unique to games where players can “cash out” profits in real money [65].

In summary, the RAFFLE comprises seven subscales, which have both parallels with, and distinctions from, motivations for gaming and gambling. Many of the RAFFLE’s subscales can be understood through the lens of self-determination theory, and/or as sources of “gratifications”. Both intrinsic motivations (enhancement, progression, social approval) and extrinsic motivations (profit through resale) of the individual are represented. Importantly, however, a subscale offering insights into the impact of more externally located influences (such as limited-time promotions) also emerged. All these subscales correlated significantly with multiple measures of engagement with loot boxes, but the “distraction/compulsion” subscale was most robustly linked. This measure of the extent to which a person is driven by feelings of compulsion, or a desire to escape boredom or their day-to-day life, may have clinical utility as an indicator of the risk of problematic involvement, and future research to test this possibility would be valuable, particularly given the concise nature of the subscale.

### 5.1. Strengths and Limitations

People with lived experience of loot box engagement were consulted throughout the development of the RAFFLE, ensuring the items were clear and meaningful to the target audience, and large, diverse samples from across the UK gaming community were recruited for each stage of the development and validation of the scale. Items were developed through comprehensive qualitative interviews, supplemented by insights from the literature and from an expert opinion panel, and two stages of validation were conducted. Although the model fit in the confirmatory factor analysis phase was (very) slightly below accepted standards on some measures, the resultant scale had good reliability (indicated by Cronbach’s alpha values), and very good criterion validity (indicated by strong and significant correlations with other measures of loot box engagement). Two of the subscales, likely due to being comprised of only two items each, had low reliability as evidenced by Cronbach’s alpha values of below 0.7; however, arbitrary cut-offs for these values are of debatable utility [66], and we considered these subscales important to retain, given their high face validity and interpretability. While they are brief, they address relatively straightforward motivations, so in the interests of parsimony, we did not consider adding further items. It will, however, be important to monitor how these subscales perform in subsequent studies.

While online data collection for studies 2 and 3 enabled us to collect data from a large number of participants in a relatively short space of time, it is important to note that this inevitably meant relying upon self-report measures of problematic involvement (i.e., the RLI), rather than utilising “gold standard” structured clinical interviews to evaluate participants” clinical characteristics, such as gambling disorder. In addition, the sample was comprised solely of UK adults, meaning that we cannot be sure how well the RAFFLE will perform in children or different geographical locations.

It is important to note the possibility that the complexity of the factor structure identified within the current studies is, in part, the product of large sample sizes with associated high statistical power. Scales developed with large samples can run the risk of identifying more factors than can be practically replicated with smaller samples in future work. It will be important to examine, in future work, whether the 23-item scale developed here consistently produces a seven-factor structure, or whether a simpler, higher order factor structure emerges.

### 5.2. Implications and Future Directions

The RAFFLE is, to our knowledge, the only validated scale for measuring the strength and nature of the diverse array of drivers of loot box engagement. We have presented preliminary evidence that some subscales correlate more strongly with problematic involvement: in particular, we predict that the distraction/compulsion subscale may prove to be a useful brief measure of risk; given its robust correlation with RLI scores. Whereas the RLI measures current risky involvement, it is possible that the distraction/compulsion subscale may play a useful pre-emptive role in predicting future risk. Future work should seek to confirm these hypotheses, uncovering how the psychology of loot box motivations relates to harm. Mediational analyses would be particularly beneficial to more fully understand the nuances of any relationships between the different types of motivation and risk of over-engagement. Future work could also explore whether there are differences in motivations for loot box purchasing across game types, in the same way that play motivations can vary across games [44]; and whether reasons for buying loot boxes in different games vary within and/or between individuals.

With many countries—including the UK, USA, Australia and Spain—currently investigating legislation for loot boxes, such studies have the potential to inform policy, educational and treatment interventions, enabling such interventions to be appropriately tailored to minimise any harms experienced by those prone to over-engagement with loot boxes in video games.

## Figures and Tables

**Table 1:** Summary of original pool of candidate items before cognitive interviews (left) and streamlined pool of items after cognitive interviews (right).

Original Item (55 Items)	Post-Cognitive Interview Changes
Arousal/Excitement	Because I find the animations/colours/sounds exciting when opening loot boxes
Because I enjoy watching the animations	
Because the colours and/or sounds are exciting	
For the excitement of seeing what will be inside	For the excitement of seeing what will be inside
Because it’s fun	Because it’s fun
Because I like the feeling of winning when I get something good	Because I like the feeling of winning when I get something good/rare
To help me progress in the game	To save time/skip the grind
To save time/skip the grind	
To get an item that will help me win	To get items that will be useful/give me an advantage
To try and get something useful	
To give me an advantage over other players	
So that I can get more out of the game	
To have the ability to compete with other people who are buying loot boxes	To level the playing field with others who buy loot boxes
Because I’ve had a failure or defeat in the game	Because I’ve had a failure or defeat in the game
To get items that I’m collecting	To get items that I’m collecting
To get items that I like the look of	To get items that I find visually appealing
To try and get items that will get me respect or attention	To get items that will get me respect or attention (“bragging rights”)
To get items to show off to my friends or other gamers (“bragging rights”)	
Because getting cool items makes me feel good about myself	To get items that make me feel good about myself
Because opening loot boxes is part of who I am	
So that I don’t get teased/made fun of	So that I don’t get shamed / made fun of
Because my friends encourage me to	Because my friends encourage or pressure me to
Because my friends pressure me to	
To join in with friends or other gamers	As a social activity
Because my friends do it	
To catch up with friends/others who have got ahead of me in the game	To catch up with friends/others who have got ahead of me in the game
Because I don’t want to feel left out	Because I don’t want to feel left out
To support good causes.	To support good causes.
To try and win items that I can sell on and make real money out of	To try and win items that I can make real money out of
To try and win items that I can sell on and make virtual currency out of	To try and win items that I can make gaming currency out of
For the chance of getting something really rare	To try and get something personally valuable to me
To try and get something valuable	
Because I get an urge to open them	Because I get an urge to open them
Because I just feel like it	
Because I can’t help myself	Because I can’t stop myself
To take my mind off the real world	To take my mind off the real world or my day-to-day life
To escape from day to day life	
For a boost when I feel low	To cheer myself up
To cheer myself up	
For a sense of escapism	Because I enjoy the sense of escape
Because I got something bad in the last box and want to try and get something better	Because I got something unwanted in the last box
Because I got something good in the last box and want to try and get something good again	Because I got something I wanted in the last box
Because I am bored	Because I am bored
Because I don’t want to miss the chance to get a limited time item or offer	Because I don’t want to miss the chance to get a limited time item or offer
Because I’ve seen a teaser about what might be inside	Because I’ve seen a teaser/preview about what might be inside
To support the developer	To support the developer
Because there’s something I want that I can only get from inside a loot box	Because there’s something I want that I can only get from inside a loot box
To get past a hurdle or sticking point in the game	To get past a hurdle or sticking point in the game
Because my card details are logged and it’s so easy	It’s so easy because my payment details are saved
Because it’s really hard to earn boxes from game play	Because I get better value with bundles, promotions, or offers
Because I keep getting duplicates	
Because I get better value, the more I buy	
Because they are offering good odds of getting something good	Because they are offering higher odds of getting something I want
Because I’m guaranteed something good	
Because there is an in-game event taking place.	Because there is an in-game event taking place.
Because streamers/pro-gamers buy them.	Because streamers/pro-gamers buy them.

**Table 2:** Descriptive statistics for the 39 candidate items.

Item	Mean	SD	Skewness	Kurtosis
For the excitement of seeing what will be inside	1.51	0.950	0.034	−0.918
To cheer myself up	0.99	0.895	0.562	−0.514
To try and win items that I can make gaming currency out of	0.94	0.963	0.616	−0.764
Because I got something unwanted in the last box	0.62	0.771	0.948	−0.048
Because I get better value with bundles, promotions, or offers	1.38	0.914	0.048	−0.834
Because I get an urge to open them	1.09	0.929	0.393	−0.803
To get items that I’m collecting	1.37	0.946	0.086	−0.916
Because I’ve seen a teaser/preview about what might be inside	1.05	0.885	0.317	−0.869
It’s so easy because my payment details are saved	1.40	1.071	0.134	−1.227
Because there is an in-game event taking place.	1.28	0.897	0.210	−0.727
To get items that will get me respect or attention (“bragging rights”)	0.64	0.877	1.146	0.216
To get items that make me feel good about myself	0.76	0.880	0.860	−0.254
Because I find the animations/colours/sounds exciting when opening loot boxes	0.62	0.880	1.232	0.435
To level the playing field with others who buy loot boxes	0.77	0.879	0.757	−0.566
To catch up with friends/others who have got ahead of me in the game	0.59	0.810	1.196	0.494
Because it’s fun	1.58	0.913	−0.093	−0.796
To support good causes.	0.49	0.726	1.367	1.212
To get past a hurdle or sticking point in the game	1.24	0.997	0.181	−1.110
To get items that I find visually appealing	1.21	1.064	0.298	−1.186
To try and get something personally valuable to me	0.90	0.932	0.701	−0.514
Because there’s something I want that I can only get from inside a loot box	1.61	0.937	−0.164	−0.844
Because my friends encourage or pressure me to	0.23	0.561	2.692	7.414
Because streamers/pro-gamers buy them.	0.31	0.625	2.211	4.801
To get items that will be useful/give me an advantage	1.59	0.972	−0.146	−0.954
Because I like the feeling of winning when I get something good/rare	1.56	0.964	−0.091	−0.945
Because I can’t stop myself	0.53	0.799	1.477	1.511
To take my mind off the real world or my day-to-day life	0.94	0.929	0.679	−0.472
Because I got something I wanted in the last box	1.06	0.841	0.330	−0.647
Because I’ve had a failure or defeat in the game	0.65	0.813	1.107	0.535
Because I am bored	1.00	0.887	0.539	−0.512
Because I don’t want to feel left out	0.41	0.698	1.617	1.850
Because I don’t want to miss the chance to get a limited time item or offer	1.36	0.913	0.090	−0.821
To support the developer	0.56	0.791	1.216	0.559
To save time/skip the grind	1.11	0.899	0.290	−0.834
So that I don’t get shamed/made fun of	0.12	0.407	3.804	15.069
Because they are offering higher odds of getting something I want	1.16	0.838	0.235	−0.617
As a social activity	0.56	0.802	1.308	0.918
Because I enjoy the sense of escape	0.95	0.885	0.636	−0.367
To try and win items that I can make real money out of	0.49	0.837	1.631	1.650

**Table 3:** Motivation Factor Pattern Matrix (rotated to the direct oblimin criterion).

Variable	Factor							
	1 Enhancement	2 Progression	3 Social Pressure	4 Distraction/Compulsion	5 Altruism	6 Fear of Missing Out	7 Resale	*h* ^2^
To get items that I find visually appealing	0.594	−0.236	0.043	0.020	0.217	−0.129	−0.031	0.591
To get items that make me feel good about myself	0.569	0.044	0.237	−0.079	0.070	−0.020	−0.037	0.513
To try and get something personally valuable to me	0.552	0.000	0.174	0.115	0.055	−0.092	0.024	0.418
Because it’s fun	0.510	0.104	−0.144	−0.174	0.119	0.017	0.072	0.424
Because I like the feeling of winning when I get something good/rare	0.508	0.080	−0.028	−0.162	−0.151	−0.052	0.196	0.491
For the excitement of seeing what will be inside	0.506	−0.066	−0.122	−0.209	−0.038	−0.149	0.239	0.597
To get past a hurdle or sticking point in the game	−0.099	0.824	−0.035	−0.015	0.071	−0.016	−0.044	0.663
To get items that will be useful/give me an advantage	0.103	0.594	−0.080	0.042	−0.182	−0.138	0.123	0.497
Because I’ve had a failure or defeat in the game	0.016	0.426	0.242	−0.138	0.003	0.065	−0.019	0.316
Because my friends encourage or pressure me to	0.068	−0.058	0.608	−0.025	0.017	0.042	0.166	0.480
So that I don’t get shamed/made fun of	−0.068	0.040	0.588	0.016	0.020	−0.067	0.094	0.389
Because I don’t want to feel left out	0.108	0.022	0.494	−0.125	−0.021	−0.070	−0.018	0.350
Because I am bored	−0.026	−0.001	−0.039	−0.673	0.072	−0.001	0.024	0.451
Because I can’t stop myself	−0.027	0.040	0.168	−0.623	−0.095	−0.019	−0.028	0.462
Because I get an urge to open them	0.226	−0.096	−0.059	−0.509	−0.002	−0.127	0.218	0.558
To take my mind off the real world or my day-to-day life	0.146	0.254	0.092	-0.457	0.162	−0.068	−0.077	0.505
To support good causes	0.002	0.114	−0.050	0.038	0.699	−0.064	0.105	0.509
To support the developer	0.044	−0.150	0.047	−0.075	0.574	0.001	−0.036	0.412
Because there is an in-game event taking place	−0.160	−0.064	0.038	0-.067	0.035	−0.798	−0.027	0.575
To get items that I’m collecting	0.195	0.142	0.018	0.065	0.043	−0.524	0.031	0.455
Because I don’t want to miss the chance to get a limited time item or offer	0.192	0.065	0.015	0.006	0.001	−0.508	0.033	0.412
To try and win items that I can make gaming currency out of	−0.088	0.050	0.046	−0.071	0.005	−0.074	0.738	0.592
To try and win items that I can make real money out of	0.084	−0.045	0.179	0.069	0.105	0.082	0.558	0.429
Cronbach’s alpha	0.815	0.655	0.634	0.737	0.603	0.693	0.644	
EIGENVALUE (prior to rotation)	5.879	2.238	1.705	1.410	1.310	1.073	1.028	
% Variance Explained	25.56	9.73	7.41	6.13	5.69	4.67	4.47	

Note: Coefficients greater than 0.40 are bold and retained for that factor. Percentage variance is post-rotation. *h*^2^ = communality coefficient (after extraction).

**Table 4:** Correlations (Spearman’s rho) between RAFFLE scores and other measures of loot box engagement.

RAFFLE Measure	Correlation with RLI Score	Correlation with Loot Box Spend
Total score	0.601 **	0.437 **
Enhancement subscale	0.516 **	0.365 **
Progression subscale	0.256 **	0.225 **
Social pressure subscale	0.307 **	0.278 **
Distraction/compulsion subscale	0.546 **	0.342 **
Altruism subscale	0.127 **	0.140 **
Fear of missing out subscale	0.350 **	0.246 **
Resale subscale	0.358 **	0.266 **

All n’s = 485. ** *p* < 0.0005.

**Table 5:** Summary of fit indices.

Model	*X* ^2^	df	*p* Value	SRMR	CFI	RMSEA	TLI
7 factor	18,948.52	253	*p* < 0.0001	0.066	0.89	0.08	0.87

**Table 6:** Construct and criterion-related validity and reliability metrics for RAFFLE overall and subscale scores.

RAFFLE Measure	Correlation with RLI Score	Correlation with Loot Box Spend	Cronbach’s Alpha
Total score	0.628 **	0.377 **	0.843
Enhancement subscale	0.473 **	0.324 **	0.789
Progression subscale	0.232 **	0.105 **	0.787
Social pressure subscale	0.381 **	0.176 **	0.718
Distraction/compulsion subscale	0.590 **	0.282 **	0.751
Altruism subscale	0.102 **	0.112 **	0.687
Fear of missing out subscale	0.422 **	0.257 **	0.702
Resale subscale	0.314 **	0.244 **	0.563

All n’s = 1495. ** *p* < 0.0005.

## Data Availability

Data used in this study can be found in the online Supplementary Materials, Tables S3 and S4.

## Supplementary Materials

The following are available online at https://www.mdpi.com/article/10.3390/jcm10245949/s1, Table S1: Order of, and rationale for, item removal during EFA; Table S2: Motivation Factor Structure Matrix (rotated to the direct oblimin criterion); Table S3: Data used in study 2; Table S4: Data used in study 3.
