# Supplementary material for: Development and Validation of the RAFFLE: A Measure of Reasons and Facilitators for Loot Box Engagement
Source: J Clin Med. 2021 Dec 18;10(24):5949. doi: 10.3390/jcm10245949 (PMC8707097; doi:10.3390/jcm10245949)
Supplement: Supplementary file 1 [file jcm-10-05949-s001.zip › Table S1.pdf]

Table S1: Order of, and rationale for, item removal during EFA

Step	Item	Reason for removal
1	'Because I find the animations/colours/sounds exciting'	4 cross-loadings, and no loadings above .247
2	'Because I got something unwanted in the last box'	2 cross-loadings, and no loadings above .293
3	'Because I got something I wanted in the last box	2 cross-loadings, and no loadings above .293
4	'It's so easy because my payment details are saved'	Lowest loading (.307)
5	'Because streamers/pro-gamers buy them.'	Lowest loading (.335)
NB	'For the excitement of seeing what will be inside' was flagged for removal at this point, based purely on loadings: It had the lowest loading (.349) and 2 other weak cross-loadings (<.3). However, it was retained due to expert opinion that it was a particularly vital item to be retained.	
6	'Because I've seen a teaser / preview about what might be inside'	Lowest loading (.397).
7	'Because there's something I want that I can only get from inside a loot box'	Cross loadings (of .335 and .446) and lowest primary loading
8	'To catch up with friends / others who have got ahead of me in the game'	Cross loadings (of .355 and .420)
9	'To level the playing field with others who buy loot boxes'	Cross loadings (of .434 and .305)
10	'Because they are offering higher odds of getting something I want'	Loading <.4 (.396) and cross loads (.256) on another factor
NB	NB Here there are multiple candidates for removal due to cross-loadings. Order of removal was decided based upon extent of the cross-loading, strength of the highest loading, and expert opinion of importance of items. 'To take my mind off the real world or my day-to-day life'; 'to cheer myself up'; and 'for the excitement of seeing what will be inside' were all candidates for removal at various points (steps 10-15) but were retained while other candidates were removed first, due to their high ratings by the expert panel and theoretical importance to the model.	
11	'Because I get better value with bundles, promotions, or offers'	Cross-loading (<=.32 on 2 factors, both of which have better (>5) loadings from other items. Also not rated highly by experts.
12	'As a social activity'	Cross loading (.333 and .472), and poor ratings by experts due to vagueness.
13	'Because I enjoy the sense of escape'	Cross loading on 3 factors and highest loading is now <.4. Experts rated as important, but this item is not very specific, judging by loadings; a similar item about 'taking one's mind off things' performs better.
14	'To save time /skip the grind'	<.4 at this point
15	'To get items that will get me respect or attention ("bragging rights")'	Cross loading <3 (and <5) on 2 factors.
16	'To cheer myself up'	Cross loading >.3 (and <.5) on two factors. Due to high expert ratings, we attempted to retain this item, but it remains a poor fit throughout and there are alternative, similar items retained, so it was removed.
