# Supplementary material for: Development and Validation of the RAFFLE: A Measure of Reasons and Facilitators for Loot Box Engagement
Source: J Clin Med. 2021 Dec 18;10(24):5949. doi: 10.3390/jcm10245949 (PMC8707097; doi:10.3390/jcm10245949)
Supplement: Supplementary file 1 [file jcm-10-05949-s001.zip › Table S2.pdf]

Table S2: Motivation Factor Structure Matrix (rotated to the direct oblimin criterion).

Variable	Factor						
	1	2	3	4	5	6	7
For the excitement of seeing what will be inside	**.695**	.068	.102	-.422	.164	-.438	.481
To get items that I find visually appealing	**.679**	-.207	.172	-.185	.441	-.362	.246
To get items that make me feel good about myself me	**.660**	.123	.380	-.353	.262	-.316	.287
Because I like the feeling of winning when I get something good / rare	**.622**	.208	.160	-.392	.015	-.337	.431
To try and get something personally valuable to me	**.609**	.040	.284	-.155	.238	-.329	.295
Because it's fun	**.602**	.154	.051	-.368	.240	-.281	.289
To get past a hurdle or sticking point in the game	-.034	**.804**	.048	-.194	-.094	-.134	-.002
To get items that will be useful / give me an advantage	.170	**.647**	.023	-.173	-.230	-.271	.206
Because I've had a failure or defeat in the game	.110	**.478**	.317	-.298	-.022	-.084	.107
Because my friends encourage or pressure me to	.248	.030	**.664**	-.211	.152	-.104	.351
So that I don't get shamed / made fun of	.118	.119	**.612**	-.156	.110	-.144	.249
Because I don't want to feel left out	.269	.137	**.550**	-.302	.109	-.203	.207
Because I am bored	.230	.163	.141	**-.667**	.137	-.175	.157
Because I can't stop myself	.198	.241	.301	**-.654**	-.012	-.168	.143
Because I get an urge to open them	.522	.094	.176	**-.627**	.167	-.380	.426
To take my mind of the real world or my day to day life	.383	.377	.278	**-.617**	.231	-.314	.163
To support good causes	.242	.007	.103	-.097	**.691**	-.232	.179
To support the developer	.208	-.218	.138	-.118	**.622**	-.113	.052
Because there is an in-game event taking place	.194	.092	.112	-.203	.166	**-.741**	.145
To get items that I'm collecting	.426	.237	.138	-.189	.171	**-.634**	.256
Because I don't want to miss the chance to get a limited time item or offer	.421	.180	.133	-.218	.143	**-.610**	.254
To try and win items that I can make gaming currency out of	.274	.150	.266	-.245	.074	-.269	**.756**
To try and win items that I can make real money out of	.308	-.018	.333	-.104	.194	-.117	**.609**
